# Treatment Options for Villous Adenoma of the Ampulla of Vater

**DOI:** 10.1155/2000/86476

**Published:** 2000-01

**Authors:** C. Hoyuela, E. Cugat, E. Veloso, C. Marco

**Affiliations:** 1 HPB Surgery Department of Surgery Hospital Mútua de Terrassa University of Barcelona Terrassa (Barcelona) Spain; 2 Pl. Dr. Robert 5 Terrassa (Barcelona) 08221 Spain

## Abstract

*Introduction:* Duodenal villous adenoma arising from the ampulla of Vater has a high risk of malignant development. Excluding associated malignant disease prior to resection of an adenoma of the ampulla is not always possible. Therefore, the surgical procedure of choice to treat this rare tumour is still controversial.

*Objective:* To evaluate retrospectively results of treatment of villous adenoma arising from ampulla of Vater with dysplasia or associated carcinoma limited to the ampulla.

*Patients and Methods:* From 1985 to 1996, eight patients have been diagnosed with ampullary villous adenoma suitable for resection. We have reviewed treatment, morbidity, mortality, follow-up and final outcome.

*Results:* Pancreatoduodenectomy (PD) was performed in 4 patients. Transduodenal ampullectomy and endoscopic resection was performed in 2 patients each. There was no perioperative mortality. None of the patients had biliary, pancreatic or intestinal leakage but two patients who underwent PD had minor postoperative complications. The mean follow-up was 44 (range: 6–132) months. Villous adenoma was associated with adenocarcinoma in 50% of the cases (4/8 patients). During the followup both patients who underwent transduodenal ampullectomy developed recurrent disease. All patients initially treated by PD are alive without evidence of recurrent disease.

*Conclusions:* Treatment of villous adenoma of the ampulla must be individualized within certain limits. In our series, PD achieve good results and it appears to be the procedure of choice in order to treat villous adenomas with proved presence of carcinoma, carcinoma *in situ* or severe dysplasia. Endoscopic or local resection may be appropriate for small benign tumours in high risk patients.


*HPB Surgery*, 2000, Vol. 11, pp. 325-331
Reprints available directly from the publisher
Photocopying permitted by license only

© 2000 OPA (Overseas Publishers Association) N.V.
Published by license under
the Harwood Academic Publishers imprint,
part of The Gordon and Breach Publishing Group.
Printed in Malaysia.

*Clinical Series*

### Treatment Options for Villous Adenoma of the Ampulla of Vater

C. HOYUELA*, E. CUGAT, E. VELOSO and C. MARCO

*HPB Surgery, Department of Surgery, Hospital Mútua de Terrassa, Terrassa (Barcelona), University of Barcelona, Spain*

*(Received 1 November 1997; In final form 21 December 1998)*

***Introduction*: Duodenal villous adenoma arising from the ampulla of Vater has a high risk of malignant development. Excluding associated malignant disease prior to resection of an adenoma of the ampulla is not always possible. Therefore, the surgical procedure of choice to treat this rare tumour is still controversial.**
***Objective*: To evaluate retrospectively results of treatment of villous adenoma arising from ampulla of Vater with dysplasia or associated carcinoma limited to the ampulla.**
***Patients and Methods*: From 1985 to 1996, eight patients have been diagnosed with ampullary villous adenoma suitable for resection. We have reviewed treatment, morbidity, mortality, follow-up and final outcome.**
***Results*: Pancreatoduodenectomy (PD) was performed in 4 patients. Transduodenal ampullectomy and endoscopic resection was performed in 2 patients each. There was no perioperative mortality. None of the patients had biliary, pancreatic or intestinal leakage but two patients who underwent PD had minor postoperative complications. The mean follow-up was 44 (range: 6-132) months. Villous adenoma was associated with adenocarcinoma in 50% of the cases (4/8 patients). During the follow-up both patients who underwent transduodenal ampullectomy developed recurrent disease. All patients initially treated by PD are alive without evidence of recurrent disease.**
***Conclusions*: Treatment of villous adenoma of the ampulla must be individualized within certain limits. In our series, PD achieve good results and it appears to be the procedure of choice in order to treat villous adenomas with proved presence of carcinoma, carcinoma *in situ* or severe dysplasia. Endoscopic or local resection may be appropriate for small benign tumours in high risk patients.**

*Keywords:* Villous adenoma, ampulla vater, pancreatoduodenectomy

### INTRODUCTION

Adenomas of the ampulla are rare neoplasms with an incidence of 0.04-0.12% in post-mortem series [1, 2]. Thereby, the clinical aspects of adenoma of ampulla are not well known because

*Address for correspondence: Pl. Dr. Robert 5, 08221 Terrassa (Barcelona). Spain. Tel.: 34.93.736.50.50, Fax: 34.93.736.50.59

of the lack of large series. These adenomas may bleed and cause recurrent pancreatico-biliary complications including jaundice, cholangitis and pancreatitis [3, 4], but the most important issue of these tumours is their potential to develope malignant changes [1, 2] and, probably, this is the main problem in deciding appropriate treatment for these tumours. Even with an accurate preoperative study, differentiating between benign adenoma and infiltrating carcinoma prior to resection is not always possible with reliability. Neither clinical presentation nor size of the lesion correlate with the presence of associated carcinoma. Endoscopy permits cholangiography, pancreatography and direct biopsies as indicated [5] but, nevertheless, excluding a carcinoma may be difficult in endoscopic biopsy specimens [6–9] because of its low reported sensitivity (42%) and specifity (79%) for detecting malignancy [6]. Even intraoperative frozen-section analysis of resected specimens may fail to detect carcinoma in 14–33% of cases [4, 10]. Therefore, it is not uncommon that the surgeon cannot rely on results of the frozen-section study during operation [8]. Endoscopic ultrasound (EUS) can provide helpful information in assessing depth of penetration [6, 11, 12] for local staging of ampullary tumours but is less accurate determining the presence or absence of lymph node metastases [11] and it cannot replace histologic evaluation [6].

Due to the high risk of association of invasive carcinoma and villous adenoma, the surgical procedure of choice to treat this rare tumour is still controversial [5, 6, 13, 14]. Pancreatoduodenectomy (PD), transduodenal excision and endoscopic resection have been reported as therapeutic options, but each approach has disadvantages. The aim of this study has been to evaluate retrospectively results of the treatment of those patients who have been diagnosed of villous adenoma of the papilla of Vater with dysplasia or early associated carcinoma at our Institution. We have reviewed treatment, morbidity, mortality, follow-up and final outcome.

### PATIENTS AND METHODS

From May 1985 to October 1996, 8 patients (4 men and 4 women) have been diagnosed with ampullary villous adenoma suitable for resection. The patients ranged from 39 to 72 years of age with a mean of 59 years. None of them had polyposis syndrome. Diagnosis was made on the basis of endoscopy and biopsy in all patients. Only those patients with histologically confirmed areas of villous adenoma with or without foci of adenocarcinoma have been included in this study. No endoscopic ultrasonography (EUS) was performed in any case. US and CT scan showed no evidence of metastatic disease. The clinical presentation, size, preoperative diagnosis, operation, final pathologic diagnosis and outcome are summarized in Table I. Most common manifestations of disease were jaundice (7/8 patients: 87.5%) and abdominal pain (3/8 patients: 37.5%). Preoperative biopsy showed focal adenocarcinoma coexisting with areas of villous tumour in 2/8 cases (25%) and severe cellular atypia in 2 cases. In 1 patient preoperative biopsy could not diagnose villous adenoma with severe dysplasia correctly. Treatment decision was taken on the basis of endoscopy (ERCP) and biopsy, size and extent of the lesion along CBD and pancreatic duct and medical status of the patient (Tab. II).

### RESULTS

Local resection was performed in 4 patients: transduodenal ampullectomy in 2 patients and endoscopic resection in 2 cases. The other 4 patients, including those with associated carcinoma, underwent pancreatoduodenectomy (PD) (2 Whipple's procedure and 2 pylorus-preserving PD). Intraoperative frozen-section studies were performed in all patients but no additional data to preoperative study were obtained in any case. Moreover, intraoperative frozen study were unable to find malignancy in one patient with infiltrating carcinoma. This patient was

TABLE I Clinical features and outcome of the patients of the series: (VA: villous adenoma; PD: pancreatoduodenectomy; M: male.; F: female)

| Case | Age/Sex | Symptoms | Size | Preop pathological diagnosis | Treatment | Postop. pathological diagnosis | Follow-up | Outcome |
|---|---|---|---|---|---|---|---|---|
| 1 | F 56 | jaundice pain | 5 cm. | Adenoca. on VA | Whipple procedure | Infiltrating adenoca. | 132 months | No recurrence |
| 2 | F 70 | jaundice | 2 cm. | Severe dysplasia | Ampullectomy | Infiltrating adenoca. | 18 months | Local recurrence at 6 m. Followed by PD. Dead from liver metastases. |
| 3 | F 72 | jaundice | 1.5 cm. | Moderate dysplasia | Endoscopic resection | Moderate dysplasia | 6 months | No recurrence |
| 4 | F 39 | jaundice pain | 3 cm. | Moderate dysplasia | PD (Pylorus-preserving) | Severe dysplasia | 84 months | No recurrence. Acute pancreatitis 2 years thereafter. |
| 5 | M 60 | jaundice | – | Moderate dysplasia | Endoscopic resection | Ca *in situ* | 24 months | No recurrence |
| 6 | M 48 | Pancreatitis | 3 cm. | Adenoca. on VA | Whipple's procedure | Infiltrating adenoca. | 43 months | No recurrence |
| 7 | M 62 | jaundice pain | 3 cm. | Severe dysplasia | Ampullectomy | Severe dysplasia | 36 months | Local recurrence at 15 months followed by PD. No recurrence |
| 8 | M 65 | jaundice | 1.5 cm. | ampullary mass | PD (Pylorus-preserving) | Severe dysplasia | 9 months | No recurrence |

TABLE II Criteria for treatment decision in the series

| |
|---|
| Endosopic resection: |
| · High-risk patients (ASA IV) (1 pat.) |
| · Tumour diameter < 1 cm. (1 pat.) |
| Local resection: (2 pat.) |
| · No evidence of malignancy |
| · Ampullary tumour without extension along CBD or pancreatic duct |
| · Tumour diameter < 3 cm. |
| Pancreatoduodenectomy: |
| · Villous adenoma with associated carcinoma (2 pat.) |
| · Tumour extension along CBD (1 pat.) |
| · Undetermined diagnosis (1 pat.) |

treated by ampullectomy and he refused a second procedure when definitive postoperative diagnosis was done (Case 2/Tab. I). Postoperative pathological report confirmed initial diagnosis in 4 cases (50%) only. One carcinoma *in situ* and 1 infiltrating carcinoma were found at final pathological examination in 2 patients with preoperative diagnosis of benign villous adenoma (Tab. I). Margins of the resected specimens were free of tumour in all patients. No lymph node metastasis were present in any case initially treated by PD.

There was no perioperative mortality. Morbidity (2/8 patients: 25%): 1 case of delayed gastric emptying after a pylorus-preserving PD procedure, 1 urinary tract infection and 1 wound infection. None of the patients had biliary, pancreatic or intestinal leakage. The mean follow-up was 44 (range: 6–132) months. The two patients who underwent transduodenal ampullectomy were presented with recurrent disease at 6 and 15 months after surgery (Tab. I). PD were then performed in both cases and pathological report of the specimen found infiltrating adenocarcinoma (1 patient died 18 months after first procedure because of hepatic metastases and the other one is without evidence of disease progression 21 months after PD). All patients initially treated by PD are alive without evidence of recurrent disease.

### DISCUSSION

Reported rates of neoplastic malignant changes in duodenal villous adenoma are high: 20–47% [1, 5, 7, 13]; previous studies have found residual adenoma at the periphery of carcinoma of the ampulla [2]. Such evidence supports the adenoma-carcinoma sequence of the ampulla of Vater and we agree with others who have recommended that all villous adenomas of the duodenum be considered potentially malignant until proven otherwise [7, 13, 14].

The major focus of controversy in the management of villous tumours of the ampulla relates to the appropriate type of resection for adenomas without invasive carcinoma. Is it necessary to perform a radical procedure as PD? Can wide local excision as transduodenal ampullectomy provide adequate resection margins? Treatment must achieve total excision of the mass in order to avoid recurrent disease because the most important determinant of survival following surgical treatment of an ampullary carcinoma is complete resection [15]. The extent of the appropriate resection is determined by size, histologic characteristics and location of the mass [8]. We share the opinion that PD remains the procedure of choice in treating adenomas containing invasive cancer [5, 13, 14]. However, the best surgical approach for benign lesions (villous and tubulovillous adenomas, and specially those with carcinoma *in situ*) confined to the ampulla is less clear [5, 6, 12–14, 16]. PD provides local control of both extensive benign lesions and cancers without distant metastases [7, 14] but several reports have advocated local ampullary resection for patients with benign and premalignant tumours and for selected patients with malignant lesions [6, 18, 16] because local excision has had good results in terms of morbidity, mortality and long-term survival. Therefore, wide local excision as ampullectomy has been proposed as the treatment of choice for these type of lesions [3, 5, 6, 8].

Nevertheless, can wide local resection of the ampulla be considered an adequate procedure? Ampullectomy is limited in tumours arising from or extending along the distal biliary or pancreatic ducts, since it may not be possible to obtain a normal proximal ductal margin. One can generally resect the pancreatic and bile ducts for 1 cm proximal to the ampulla [6,14]. Furthermore, it can be difficult to reconstruct larger duodenal mucosal defects if the size of the tumour is greater than 3 cm [6]. In our series, one patient underwent pylorus-preserving PD in order to treat a villous adenoma without signs of malignancy due to its proximal extension along common bile duct (case 4/Tab. I). Moreover, recurrence of a benign adenoma after adequate local removal is nonetheless uncommon [5,7,13]. The recurrence rate of local excision in treating villous adenomas was 28% in Cleveland Clinic's series [13]. In the same report, 62% of the patients treated by duodenotomy and wide local excision had postoperative complications, against 40% of those patients treated by PD. In 64 patients collected from different reports who underwent local resection of any ampullary carcinoma, overall mortality rate was 6% [15]. If local resection has always been a tempting alternative for the treatment of small ampullary tumours due to the theoretical advantage of avoiding mortality and morbidity of PD, this advantage is not very clear. Transduodenal wide local excision of the ampulla cannot be considered a minor procedure.

In the past, PD has been associated with a high morbidity and mortality. However, large series have documented more recently that complication rates associated to PD are decreasing because of improved perioperative care [13, 17, 18]. There were no deaths in a recent series of 142 consecutive patients who underwent PD [18]. Morbidity and mortality following an aggressive approach such PD have been low in our series too. The pylorus-preserving modification of the Whipple's procedure [19] is actually our procedure of choice because it is an appropriate oncological procedure and it reduces the incidence of troublesome post-gastrectomy symptoms with better preserved gastro-intestinal function [20].

The endoscopic approach has been proposed as the least invasive approach to remove an ampullary adenoma [21,22]. However, local recurrence after limited excision is a hazard [21] because early carcinoma could be missed if the lesion is not totally excised [3]. In a series of 25 patients treated by endoscopic snare excision tumour recurred in 26% (mean follow-up 37 months). In our opinion, endoscopic approach should be used only in patients who are at high risk for medical reasons. It may be used also to palliate some of the symptoms, as jaundice or recurrent cholangitis [3,9].

In conclusion, treatment of villous adenoma of the ampulla must be individualized within certain limits. The size, extent of the lesion, the presence of a carcinoma and the medical status of the patient must be evaluated. But, considering the potentially high risk of malignancy of villous adenoma of the ampulla of Vater and the uncertainty of obtaining accurate pathologic diagnosis before surgery, PD appears to be the procedure of choice in order to treat villous adenomas with proved presence of carcinoma, carcinoma *in situ* or severe dysplasia. PD provides local control with acceptable morbidity of both extensive benign lesions and cancers without distant metastases and it achieves good results in our series. Endoscopic or local resection may be appropriate for small benign tumours in high risk patients. If an infiltranting carcinoma is found or if margins are involved, the procedure should be converted to a PD.

### *References*

[1] Yamaguchi, K. and Enjoji, M. (1991). Adenoma of the ampulla of Vater: putative precancerous lesion. *Gut*, **32**, 1558-1561.
[2] Yamaguchi, K. and Enjoji, M. (1987). Carcinoma of the ampulla of Vater. A clinicopathologic study and pathologic staging of 109 cases of carcinoma and 5 cases of adenoma. *Cancer*, **59**, 506–515.

[3] Sand, J. A. and Nordback, I. H. (1995). Transduodenal excision of benign adenoma of the papilla of Vater. *Eur. J. Surg.*, **161**, 269–272.

[4] Sharp, K. W. and Brandes, J. L. (1990). Local resection of tumors of the ampulla of Vater. *Am. Surg.*, **58**, 214–217.

[5] Bjork, K. J., Davis, C. J., Nagorney, D. M. and Mucha, P. (1990). Duodenal villous tumors. *Arch. Surg.*, **125**, 961–965.

[6] Rattner, D. W., Fernández-del Castillo, C., Brugge, W. R. and Warshaw, A. L. (1996). Defining the criteria for local resection of ampullary neoplasms. *Arch. Surg.*, **131**, 366–371.

[7] Ryan, D. P., Schapiro, R. H. and Warshaw, A. L. (1986). Villous tumors of the duodenum. *Ann. Surg.*, **203**, 301–306.

[8] Asbun, H. J., Rossi, R. L. and Munson, J. L. (1993). Local resection for ampullary tumors. Is there a place for it? *Arch. Surg.*, **128**, 515–520.

[9] Shemesh, E., Nass, S. and Czerniak, A. (1989). Endoscopic sphincterotomy and endoscopic fulguration in the management of adenoma of the papilla of Vater. *Surg. Gynecol Obstet.*, **169**, 445–448.

[10] Chappuis, Ch. W., Divicenti, F. C. and Cohn, I. (1989). Villous tumours of the duodenum. *Ann. Surg.*, **209**, 593–599.

[11] Mukai, H., Nakajima, M., Yasuda, K., Mizuno, S. and Kawai, K. (1992). Evaluation of endoscopic ultrasonography in the preoperative staging of carcinoma of the ampulla of Vater and common bile duct. *Gastrointest Endosc.*, **38**, 676–683.

[12] Tio, T. L., Mulder, C. C. J. and Eggink, W. F. (1992). Endosonography in staging early carcinoma of the ampulla of Vater. *Gastroenterology*, **102**, 1392–1395.

[13] Galandiuk, S., Hermann, R. E., Jagelman, D. G., Fazio, V. W. and Sivak, M. V. (1988). Villous tumours of the duodenum. *Ann. Surg.*, **207**, 234–239.

[14] Targarona, E. M., García-Olivares, E., Fernández-Trigo, V., Muñoz, E., González, G., Forcada, P. *et al.* (1990). Duodenal villous adenoma. *Cir. Esp.*, **47**, 365–374.

[15] Allema, J. H., Reinders, M. E., van Gulik, T. M., van Leeuwen, D. J., Verbeek, P. C. M., de Wit, L. T. and Gouma, D. J. (1995). Results of pancreaticoduodenectomy for ampullary carcinoma and analysis of prognostic factors for survival. *Surgery*, **117**, 247–253.

[16] Gertsch, P. H., Matthews, J. B., Lerut, J., Bauer, H. U. and Blumgart, L. H. (1990). The technique of papilloduodenectomy. *Surg. Gynecol Obstet.*, **170**, 254–256.

[17] Cameron, J. L., Pitt, H. A., Yeo, C. J., Lillemoe, K. D., Haufman, H. S. and Coleman, J. A. (1993). One hundred and forty-five consecutive pancreatiocoduodenectomies without mortality. *Ann. Surg.*, **217**, 430–438.

[18] Fernández del Castillo, C., Rattner, W. and Warshaw, A. L. (1995). Standards for pancreatic resections in the 1990's. *Arch. Surg.*, **130**, 295–299.

[19] Traverso, L. W. and Longmire, W. P. Jr. (1978). Preservation of the pylorus in pancreaticoduodenectomy. *Surg. Gynecol Obstet*, **146**, 959–962.

[20] Kozuschek, W., Reeth, H. B., Waleczeck, H., Haarman, W., Edelmann, M. and Sonntag, D. A. (1994). Comparison of long-term results of the standart Whipple procedure and the pylorus-preserving pancreaticoduodenectomy. *J. Am. Coll. Surg.*, **178**, 443–453.

[21] Binmoeller, K. F., Boaventura, S., Ramsperger, K. and Soehendra, N. (1993). Endoscopic snare excision of benign adenomas of the papilla of Vater. *Gastrointest. Endosc.*, **39**, 127–131.

[22] Lambert, R., Ponchon, T., Chavillion, A. and Berger, F. (1988). Laser treatment of tumours of the papilla of Vater. *Endoscopy*, **20**, 227–233.

### COMMENTARY

This interesting case series of eight patients illustrates several facets of the management of villous adenomas of the duodenum. The treatment of this disease is influenced by emerging facts regarding its natural history as well as advances in endoscopic surgery and open surgery. Of key importance is the fact that about 40% of villous adenomas actually contain invasive ampullary cancer (3 of 8, or 38% in this series). Recurrence after surgical ampullectomy has been frequently reported and happened in both patients in this series. Surgical ampullectomy is also limited by the size of the tumor. Endoscopic methods are minimally invasive and can completely excise or destroy small benign lesions. They also provide close follow-up after excision and close follow-up with biopsy is an integral part of local excision. Pancreaticoduodenectomy has emerged now as a safe procedure with a mortality rate of equal to, or less than, 1% in specialized HPB centers. Importantly, the quality of life after pancreaticoduodenectomy is very high and equivalent to that of cholecystectomy.

Given these facts, pancreaticoduodenectomy must be considered the treatment of choice for a villous adenomas that contain invasive cancer, cancer *in situ*, or in which the pathology is uncertain. Pancreaticoduodenectomy is also indicated for lesions larger than 3 cm. in diameter. For smaller lesions, particularly in high risk patients, local excision becomes competitive with pancreaticoduodenectomy. It should be noted that the oldest patient in this series is 72 years. Therefore all patients were well within the age range in which pancreaticoduodenectomy is commonly performed very safely.

In small lesions patient input is particularly important.

Pancreaticoduodenectomy offers the greatest chance of long-term cure, but while safe, is a very invasive procedure. When choosing between the type of local excision, endoscopic excision and close follow-up is probably as effective in skilled hands as ampullectomy, and is much less invasive. As a result, ampullectomy is being used less and less.

Dr. S. M. Strasberg
Department of Surgery
School of Medicine, Washington University
One Barnes Hospital Place
St. LOUIS
Mo 63110
USA

